# Community use of systemic antibiotics among individuals aged 15 and over in Brazil: A seven-year population-based cross-sectional study

**DOI:** 10.1371/journal.pone.0325231

**Published:** 2025-06-05

**Authors:** Tatiana de Jesus Nascimento Ferreira, Rosângela Caetano, Ria Benkö, João Henrique de Araújo Morais, Claudia Garcia Serpa Osorio-de-Castro

**Affiliations:** 1 Department of Medicines Policies and Pharmaceutical Services, Sergio Arouca National School of Public Health - Oswaldo Cruz Foundation, Rio de Janeiro, Brazil; 2 Department of Health Policy, Planning and Administration, Institute of Social Medicine, Rio de Janeiro State University, Brazil; 3 Institute of Clinical Pharmacy, University of Szeged, Szeged, Hungary; 4 Postgraduate Program in Public Health Epidemiology, Sergio Arouca National School of Public Health - Oswaldo Cruz Foundation, Rio de Janeiro, Brazil; Lahore Medical and Dental College, PAKISTAN

## Abstract

Brazil is recognized as the largest consumer of antibiotics among Latin American countries, despite the implementation of restrictive measures since 2011. Systemic antibiotics (J01) are commonly prescribed for community use and empirically for treating viral diseases, which can result in therapeutic failure and potential sources of microbial resistance. Studying the use of J01 at the outpatient and community level provides an opportunity to understand different clinical and social perspectives on the use of these drugs. The study aimed to describe the consumption of J01 in young people and adults in Brazil, based on dispensing from private community pharmacies. We conducted a cross-sectional study using data from January 2014 to December 2020, extracted from dispensing records in the National System for the Management of Controlled Products at the national, regional, and state levels. The primary consumption indicator used was the Defined Daily Dose per 1,000 inhabitants per day (DID). A total of 259,313,837 antibiotic dispensing records were collected during the period. Of this total, 67.2% were J01 and complied with other inclusion criteria established for the analysis. Over the period, 4,590,329,296 standard units were consumed in Brazil, characterized by a non-linear trend (p-value 0.357). Consumption ranged from 9.8 to 12.9 in DID. Penicillins (J01C) and macrolides (J01F) were the most consumed therapeutic groups, accounting for 28.1% and 28.6% of total J01 consumption, respectively, in terms of median usage. The analysis revealed that although overall consumption is increasing across the country, the patterns differ based on the distribution of dispensing records and DID values in various states. The results provide insights that can serve as a foundation for local health managers to analyze and interpret the data, promoting the development of surveillance and monitoring strategies for the use of J01.

## Introduction

The discovery and introduction of antibiotics in the first half of the twentieth century was undoubtedly one of the greatest medical advances of our time [1]. The use of these drugs in humans and animals has provided effective treatments for several infectious diseases and increased life expectancy. However, excessive and inappropriate use of antibiotics increases the development of antimicrobial resistance (AMR) [2]. Moreover, gaps in discovering and launching new antibiotics make the crisis more severe due to a lack of appropriate treatment options [3]. Inasmuch, studies of the magnitude and trends of consumption of these drugs are critical for public health and clinical considerations, especially in low-and middle-income countries (LMICs), where AMR has been consistently linked to mortality rates, mainly due to lack of awareness [4,5].

Brazil is a vast upper-middle-income country of continental proportions, having the largest population in Latin America [6]. The 2022 census established a population of 207,750,291 inhabitants [7], mainly urban. The country is divided into five regions (North, Northeast, Southeast, South, and Midwest) comprising 26 states and the Federal District (Brasília), the country’s capital [8]. Between 2000 and 2010, Brazil presented the largest antibiotic consumption in Latin America [9,10]. Additionally, consumption from 2013 to 2016 showed a relative growth of 18%, with greater growth concentrated in the North and Northeast regions, which have a lower overall income [11].

Since 2010, the dispensing of antimicrobials – a broad term, that includes not only antibiotics (substances with activity against bacteria), but also antivirals, antimycotics and antiparasitic products – in Brazil has been federally regulated, permitting pharmacy dispensing only upon presentation of a prescription issued by a medical professional, veterinarian, or dentist [12]. The National Controlled Products Management System (SNGPC), administered by the Brazilian Health Regulatory Agency (ANVISA), plays a key role [13], storing private pharmacy dispensing data for medicines subject to special control, such as central nervous system medications and antimicrobials. This System is not linked to the Brazilian Unified Health System (SUS) public pharmacies, which dispense drugs free of charge.

As of August 2020, ANVISA allowed public and unrestricted access to data entered in the SNGPC since 2014 for consultation and independent research on a specific site organized into two blocks: medicines for the central nervous system and antimicrobials. Previous studies were subject to prior authorization and receipt of data by the agency [11,14–16].

This study analyzed community consumption of J01 in Brazil between 2014 and 2020 at national and state levels.

## Method

### Research design

We conducted a retrospective longitudinal study using dispensing data from private community pharmacies in Brazil over seven years from January 2014 to December 2020.

### Data source

All dispensing records linked to the antibiotics group, publicly available on the ANVISA website, were downloaded directly from the specific website.

### Data extraction

Dispensing records from private community pharmacies containing data on a single drug dispensed on a prescription for human use, used as monotherapy or as a fixed-dose combination, such as antibiotics with enzyme inhibitors (e.g., amoxicillin with clavulanic acid) or two antibiotics (e.g., trimethoprim-sulfamethoxazole), were extracted. Each dispensing record extracted refers to a single dispensed J01medicine (in a fixed-dose combination or not).

### Record selection

Three selection criteria were established based on our previous qualitative assessment of the SNGPC data [17]. First, all extracted antibiotic dispensed records were categorized according to the WHO Anatomical Therapeutic Chemical (ATC) classification [18]. Only J01 dispensing records were selected in this initial filtering process. We also checked for data completeness, ensuring that only J01 records containing complete information on the dosage form, strength, and quantity of antibiotic dispensing were considered at this stage. Finally, we focused on the population of interest: individuals aged 15 years or older (PopE). According to Ferreira et al. [17], this age group comprises more than 83% of the records entered the system, establishing our third selection criterion.

### Data processing

For each selected record, the following variables were included: Brazilian Common Name [19]; ATC classification from 1^st^ level (main group) to 5^th^ level (active substances); defined daily dose (DDD), based on the respective updates of the 2021 WHO version 21 [18]. In addition, the content of the active substance of the antibiotics in international units (IU) was converted to milligrams [20].

### Data analysis

To conduct a comprehensive analysis of J01 antibiotic use, we standardized consumption data based on the annual PopE estimates provided by the Brazilian Institute of Geography and Statistics [21]. We selected six metrics at both national and state levels (see Table 1) to measure the annual consumption of these antibiotics [22–24]. We assessed the time series trends at ATC level 5th for antibiotics with a monthly DID greater than or equal to 0.05 at any given time at the national level. We applied for the Student Sieve-bootstrap t-test with a significance level of 5% (α ≤ 0.05) [25]. Additionally, an Analysis of Variance (ANOVA) test was conducted on the total J01 consumption dataset and at the ATC 3^rd^ level to determine the statistical significance of consumption from 2014 to 2020, using a significance level of 5% (α ≤ 0.05). For all descriptive statistical analyses and calculation of consumption measures, we utilized the statistical package R, version 4.0.2.

**Table 1:** Metrics selected to measure the consumption of systemic antibiotics in Brazil and its states, 2014-2020.

Consumption proxy indicators	Analysis dimension	Metrics	Description	Level/period	Calculation
Accessibility^1^	(i) Total J01 dispensing records per inhabitant/year	The sum of J01 dispensing records per inhabitant each year.	State annual	$\frac{total\;number\;of\;J01\;dispensing}{popE}$
Uso	(ii) Utilization in Dose Daily Dose (DDD)^2^ per inhabitant	The sum of utilization in DDD per inhabitant^2^	State annual	$\frac{(number\;of\;packages\;dispensingx\;(number\;of\;DDD\;in\;packages}{popE}$
	(iii) DDD per 1000 inhabitants per day (DID)^2^	Number of defined daily doses (DDD) per 1,000 inhabitants per day (DID) by J01	State and national annual	$\frac{Sum\;utilization\;in\;DDD\;dispensed\;by\;J01\;}{popE\;x\;365}x\;1000$
		Number of defined daily doses per 1,000 inhabitants per day (DID) by ATC 3^rd^ level	National annual	$\frac{total\;DDDs\;dispensed\;by\;ATC\;3th\;level\;}{popE\;x\;365}x\;1000$
		Number of defined daily doses per 1,000 inhabitants per day (DID) by ATC 5^th^ level	National monthly	$\frac{total\;DDDs\;dispensed\;by\;ATC\;5th\;level\;}{popE\;x\;30}x\;1000$
	(iv) Compound Annual Growth Rate (CAGR)^3^	Mean annual change as a proportion (%) of consumption in the first year until the last year of the period	State and national annua	[(DIDend/DIDstart)^(1/N)]−1
	(v) Relative consumption of ATC 3rd level	Relative consumption of the ATC 3^rd^ level of total J01 consumption	National annual	$\frac{absolute\;consumption\;of\;the\;ATC\;3rd\;level\;by\;state\;}{absolute\;national\;consumption\;of\;J01l}x100$
	(vi) Relative consumption of the major ATC 3rd level	Relative consumption of the major ATC 3^rd^ level	State	$\frac{absolute\;consumption\;of\;the\;ATC\;3rd\;level\;by\;state\;}{absolute\;national\;consumption\;of\;3rd\;level}x100$

Source: Study dataset, 2022.

Note:

^1^Affordability comprises a measure of barriers or facilitators to access [26].

^2^DDD and DID indicators were calculated using the WHO Anatomical Therapeutic Chemical (ATC) classification system as recommended by the World Health Organization (WHO, 2021) [18]

^3^CAGR was calculated as described in the works of Robert et al. and Mthombeni et al. [23–24]

## Results

**Fig 1:**
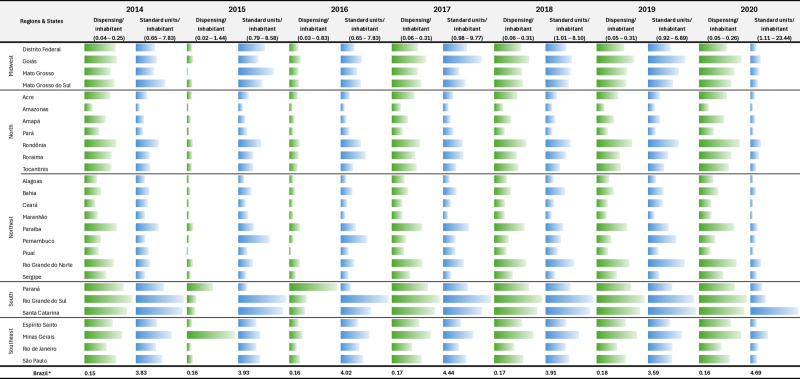
Consumption of systemic antibiotics measured by the number of dispensing records^1^ and number of standard units dispensed per inhabitant^2^ according to the state^3^, Brazil, 2014-2020. Source: Study dataset, 2022. Notes: ^1^(n): Total number of dispensing records for antibiotics for systemic use (ATC J01) per inhabitant in each year of the study of all 27 states (Brazil). ^2^Total standard units dispensed per inhabitant in each year of the study of all the 27 states (Brazil). ^3^Brazilian States according to geographic region: Midwest: Mato Grosso (MT), Goiás (GO), Brasília, Federal District (DF), Mato Grosso do Sul (MS). North: Tocantins (TO), Pará (PA), Amapá (AP), Roraima (RR), Amazonas (AM), Acre (AC), Rondônia (RO). Northeast: Bahia (BA), Sergipe (SE), Alagoas (AL), Pernambuco (PE), Paraíba (PB), Rio Grande do Norte (RN), Ceará (CE), Piauí (PI), Maranhão (MA). South: Rio Grande do Sul (RS), Santa Catarina (SC), Paraná (PR). Southeast: São Paulo (SP), Minas Gerais (MG), Rio de Janeiro (RJ), Espírito Santo (ES).

A total of 259,313,837 antibiotic dispensing records spanning January 2014 to December 2020 were retrieved. Of these, 185,351,185 (71.5%) met the inclusion criteria: records of antibiotics of the ATC J01 group containing complete information on the antibiotic and prescribed to individuals aged 15 years or over. Eighty-eight percent of the dispensing records were prescriptions issued by doctors; the rest were dental prescriptions. Almost all dispensing records (99%) lacked information on the International Classification of Diseases (ICD-10) codes associated with the prescribed antibiotics. A total of 4,590,329,202 defined daily doses (standard units) of J01 were consumed in Brazil throughout the study period. Fig 1 shows the distribution of records and standard units dispensed per inhabitant/year by Brazilian state.

**Fig 2:**
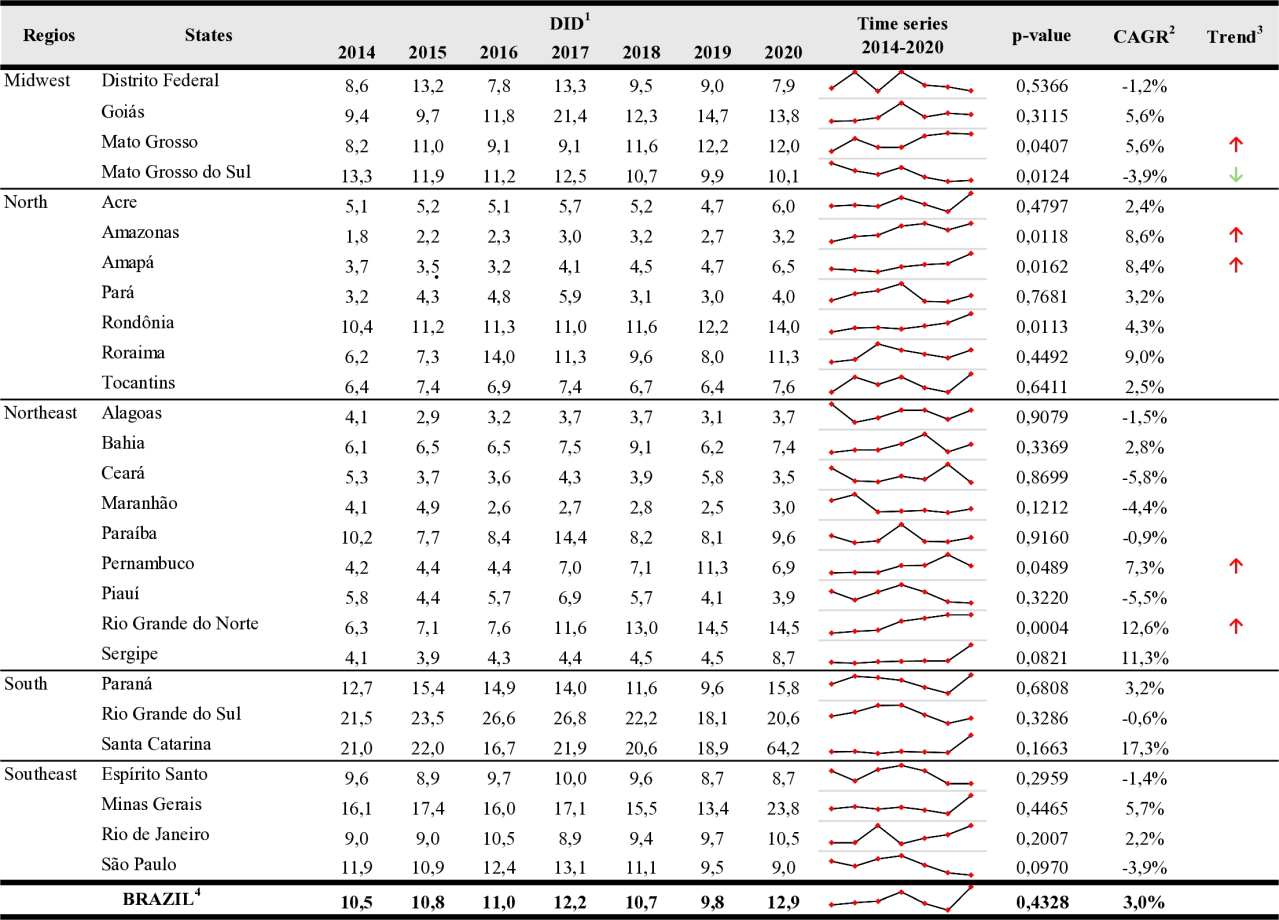
Community consumption of J01 antibiotics at national and state levels expressed as DDD per 1000 inhabitants and per day (DID), Brazil, 2014−2020. Source: Study dataset, 2022. Note: ^1^DID refers to the DDD per 1000 inhabitants per day. ^2^CAGR = [(DIDend/DIDstart)^(1/N)]-1 (N = period). ^3^Trend- indicates that the change in consumption (↑ increase or ↓ decrease) was statistically significant (p-value ≤ 0.05%). ^4^ Refers to the average consumption of J01 weighted by the respective population of all 27 states (Brazil).

Fig 2 presents the community consumption of J01, measured in DID, together with the CAGR at the national and state levels. The country experienced an increase in antibiotic consumption over the years, with CAGR of 3.0%, averaging 11.1 DID (ranging from 9.8 to 12.9) and exhibiting some fluctuations without a statistically significant impact (p-value = 0.4328).

At the state level, consumption varied from 1.8 to 64.2. Statistically significant changes indicating an increase in consumption were observed in the states of Amazonas, Amapá, Rondônia, Mato Grosso, and Rio Grande do Norte. While only in Mato Grosso do Sul there was a statistically significant reduction.

The states that presented the lowest DID were Amazonas, in the first three years, and Maranhão in the last four years, both of which are in the North Region. Located at the other end of the country, in the South Region, two states presented the highest DIDs in the entire series: Rio Grande do Sul in the first six years and Santa Catarina in 2020.

**Fig 3:**
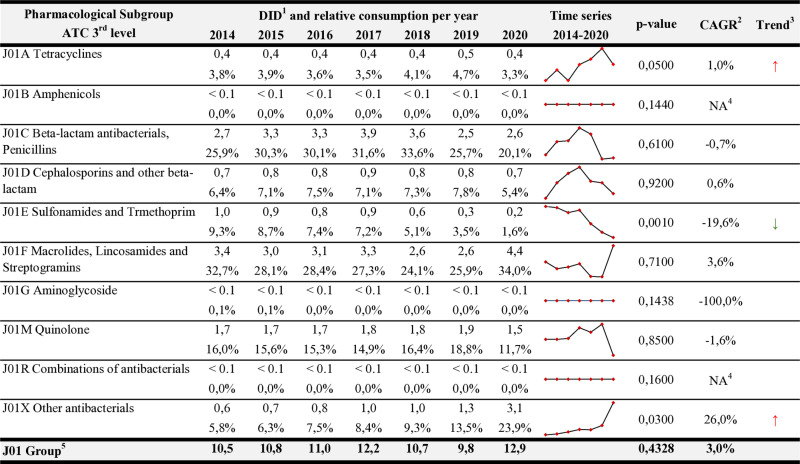
Community consumption of systemic antibiotics, according to Therapeutic Subgroup ATC 3^rd^ level, expressed as DDD per 1000 inhabitants and per day (DID) and relative consumption of total consumption, Brazil, 2014−2020. Source: Study dataset, 2022. Note: ^1^DID refers to the Defined daily doses per 1000 inhabitants and per day. ^2^CAGR = [(DIDend/DIDstart)^(1/N)]-1 (N = period). ^3^ Indicates ↑ increase or ↓ decrease in consumption statistically significant (p-value ≤ 0.05%). ^4^NA (non-applicable). Null DID values. ^5^Refers to the average consumption of J01 (ATC J01) weighted by the respective population of all the 27 states of Brazil.

Fig 3 shows the annual consumption of J01 in DID and the relative consumption according to the ATC 3rd level. Consumption ranged from 0.0 (amphenicols and combinations of antibacterials, both in the entire series) to 4.4 (macrolides, in 2020). Macrolides (J01F), penicillins (J01C), and quinolones (J01M) were the groups of antibacterials with the greatest consumption in the period, representing together more than 70% of the total consumption of antibiotics in the country. Classes that showed a statistically significant trend in consumption over the years were tetracyclines (J01A) and other antibacterials (J01X) with an increase; sulfonamides and trimethoprim (J01E) with a decrease.

In 2020, the consumption of macrolides (J01F) was 1.7 times that of 2019, and it was also the highest DID identified in the historical series (DID 4.4). In percentage terms, the therapeutic subgroups macrolides (J01F), other antibacterials (J01X), and penicillins (J01C) were the most consumed among J01: 34%, 23.9%, and 20.1%, respectively.

**Fig 4:**
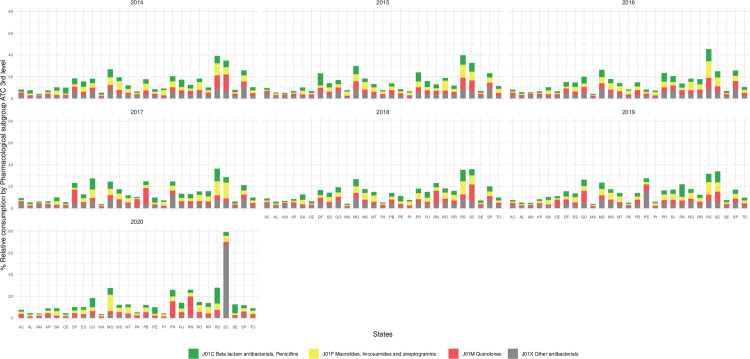
Relative annual consumption, expressed as % DDDs per 1000 inhabitants and per day (DID), of the therapeutic subgroups with the highest absolute consumption in the country (J01C, J01F, J01M and J01X), according to State^1^ of dispensing, Brazil, 2014-2020. Source: Study dataset, 2022. Note: ^1^Brazilian States according to geographic region: Midwest: Mato Grosso (MT), Goiás (GO), Brasília – the Federal District (DF), Mato Grosso do Sul (MS). North: Tocantins (TO), Pará (PA), Amapá (AP), Roraima (RR), Amazonas (AM), Acre (AC), Rondônia (RO). Northeast: Bahia (BA), Sergipe (SE), Alagoas (AL), Pernambuco (PE), Paraíba (PB), Rio Grande do Norte (RN), Ceará (CE), Piauí (PI), Maranhão (MA). South: Rio Grande do Sul (RS), Santa Catarina (SC), Paraná (PR). Southeast: São Paulo (SP), Minas Gerais (MG), Rio de Janeiro (RJ), Espírito Santo (ES).

The relative consumption of the main therapeutic subgroups (J01C, J01F, J01M, and J01X) by Brazilian states is shown in Fig 4. For penicillins (J01C), the maps show higher relative consumption in the states located in the South and Southeast regions throughout the period. Macrolides (J01F) have consumption levels similar to those of penicillins, concentrated in the South, Southeast, and Central-West regions. Quinolones (J01M) present a more widespread pattern, with high relative consumption varying in the South, Southeast, North, and Northeast regions. Finally, other antibacterials (J0IX) present less intense relative consumption, with emphasis on the atypical consumption concentrated in Santa Catarina in the last year of the series.

The year 2020 stands out for having the highest relative consumption in each of the groups in different states: Rio Grande do Sul (penicillins 14.7%); Minas Gerais (macrolides 15.1%); Paraná (quinolones 14.1%) and Santa Catarina (other antibacterials 66.9%).

**Fig 5:**
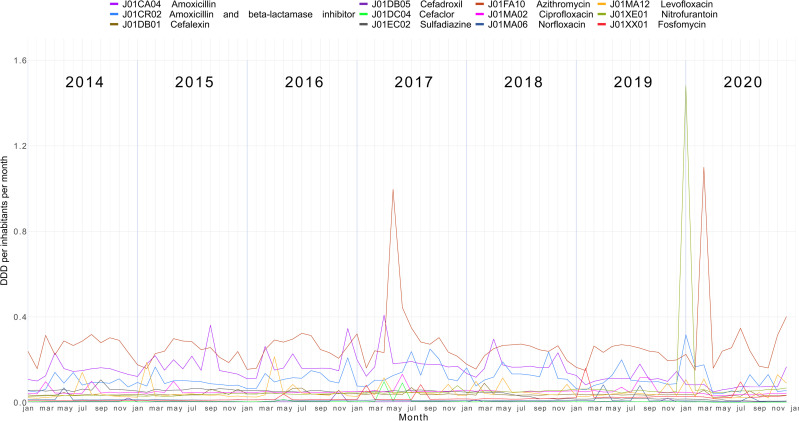
Monthly community consumption of J01 antibiotics (ATC level 5) expressed as DID*. Brazil, 2014-2020. Source: Study dataset, 2022. *Only antibiotics with a DID of ≥ 0.05- at some point throughout the time series were included.

The monthly community consumption of J01, during the period, according to ATC 5th level expressed as DID, is shown in Fig 5. Only antibiotics with monthly DID values higher or equal to 0.05 at some point in the historical series appear in the graph. The results of the bootstrap t-test indicate that consumption followed a non-linear trend during the period (p-value = 0.357). Throughout the historical series, the most consumed antibiotic was azithromycin (J01FA10), followed by amoxicillin alone (J01CA04) or in combination with clavulanic acid(J01CR02). During the period, four outliers were identified: azithromycin (J01FA10) in March 2017, March and December 2020, and nitrofurantoin (J01XE01) in January 2020.

## Discussion

This study examines the consumption of J01 over seven uninterrupted years at national and state levels, using data obtained from SNGPC but focusing on population aged => 15 years [17].

The analysis showed that consumption, which is growing and significant in some states, follows an upward trajectory in the country (CAGR 3.0%), with profiles that do not necessarily follow the size of the population in the states, both in relation to the number of dispensing records or DIDs. States with the highest number of systemic antibiotic dispensing records per inhabitant continued to have the highest number of dispensed DDDs, so population size may not be a leading factor in understanding antibiotic consumption in these states. Factors such as accessibility to health services and pharmacies, as well as family income, may have influenced this profile.

The average J01 consumption in the country during the seven-year study period was 11.1 DID, ranging from 9.8 to 12.9. When comparing J01 consumption rates with those of European countries included in the European Surveillance of Antimicrobial Consumption Network (ESAC-Net) report, which also considered pediatric data (DID ranging from 14.1 to 18.3) [27], our results demonstrate a much lower consumption. However, similar findings to ours have been observed in other countries that used a similar methodology to ESAC-Net [24,28,29].

In LMICs, such as Brazil, ensuring access to medicines requires a complex systemic approach encompassing a series of requirements, from the right to medicines to the affordability of medicines for patients and users [26]. The distribution of consumption, measured by the number of records and standard units per inhabitant per year for each state (Figs 1A and 1B), suggests possible facilitators or barriers related to access to medicines, as dispensing records are linked to prescriptions on one side and to sales on the other. Firstly, the geographic availability of healthcare facilities is an aspect to consider. Findings for both São Paulo and Minas Gerais, populous states in the country’s most populous region, the Southeast, reveal a greater density of health services [30]. Conversely, states in the North Region, which are less populated and have fewer healthcare services, tend to show less access to prescription medications.

Affordability and demand-side factors [26] are also very important. The WHO estimated that about 56% of the population in lower-middle-income countries access antibiotics in the private sector [31]. The Brazilian retail market is the 5th largest among the most populous countries [32]. The wealthy Southeast, where São Paulo, Minas Gerais, and Rio de Janeiro are located, together with Rio Grande do Sul in the South, corresponded to 53.1% of the country’s pharmaceutical retail market in 2023 [32]. In the last 20 years, large pharmacy chains have been significantly expanding in the Southeast, reflecting the country´s socioeconomic inequalities [33]. The South and the Southeast regions have the highest Gross Domestic Product (GDP) [34], thus favoring the acquisition of medicines through out-of-pocket payments. Economic factors may partially explain the greater concentration of J01 registers in states located from the Brazilian central west to the southern region [35].

Direct payment, linked to socioeconomic factors like unemployment, may be associated with consumption [36]. Bahia, the 4th most populous state in Brazil but ranked 18th in GDP [34] with the highest unemployment rate (15.5% in 2022) [35], contributed less than 5% of total dispensing records and less than 3% of total DID. A strong correlation between the unemployment rate and low consumption of J01 antibiotics has been described in Poland [37]. This correlation is directly linked to individual economic factors and local development. In Turkey, cities with lower socioeconomic development indices also had lower antibiotic consumption rates [38]. However, lower or higher consumption of J01 may not be linked to higher economic *status*. In Hungary, citizens who received regular social assistance and lived in poor sanitary conditions were found to consume more antibiotics due to free access to certain medicines or a higher incidence of community-acquired infections due to substandard housing [39].

Migration may lead to an increased demand for health services [40], especially in the northern states. Since 2015, Roraima has experienced a significant influx of immigrants from Venezuela, resulting in a higher demand for healthcare, particularly in primary care [41], as evidenced by the increased number of dispensing records and standard units. Additionally, the findings for Rondônia may also be linked to migratory trends along the Brazil-Bolivia border [42] and the border of the state of Mato Grosso. This is likely due to gaps in health services across large areas of the Brazilian Amazon [43], with statistically significant impacts reflected in a CAGR of 4.3% (Fig 2).

The annual time series revealed two peaks in overall J01 consumption in 2017 and 2020. Even if it is not possible to pinpoint the exact causes of increased consumption in 2017, a considerable number of cases of severe respiratory infections were reported in 2017 in the Northeast, South, and Southeast [44]. Furthermore, since 2016, Chikungunya virus disease (CHVD) has severely affected Brazilian states, showing a 10–20-fold increase in suspected cases in 2016 and 2017 [45]. Although it is a viral disease, it can have severe effects on the lungs, leading to pneumonia, respiratory failure, and meningitis [46]. These conditions may have been the underlying cause of the increase in consumption.

The situation in 2020 was unprecedented compared to previous years, marked by the onset of the COVID-19 pandemic. Analyzing consumption for that year may provide insights into the impact of the pandemic and social distancing policies on antibiotic prescriptions J01. In several European countries, both J01 use and community-acquired infections decreased [27,47,48], possibly due to a decrease in the consumption of penicillins (J01C by 23.0%), cephalosporins and other β-lactam antibacterials (J01D by 25.8%), and macrolides (J01F by 17.4%) [49]. In the United States, community pharmacy dispensing of antibiotics decreased by 26.8% between March and December 2020, compared with the same period in 2017 and 2019 [50]. A slight increase in azithromycin prescriptions may have occurred during the first wave in the US, but total prescriptions decreased during the pandemic [51].

In 2020, several J01 subclasses were in downfall (tetracyclines, penicillins, cephalosporins, sulfamethoxazole and trimethoprim, aminoglycosides, and quinolones) in Brazil. Brazil was not able to successfully implement measures to minimize the excessive use of J01 during the pandemic, due to inappropriate prescribing, mainly of macrolides [52,53]. Considerable numbers of prescription records with large volumes of antibiotics for individual patients were identified by Ferreira et al in the first quarter of this year [17]. This strongly suggests the accumulation of prescriptions as aggregates due to large prescription volumes being simultaneously fed into the system or pooled [17]. In the Eastern Mediterranean, COVID-19 was associated with a considerable increase in the prevalence of antibiotic self-medication, from 20.8% to 45.8%, which was mediated by pharmacist recommendations or patient requests [54].

The high consumption of macrolides (J01F), compared to penicillins, in Brazil is concerning. Throughout the study period, a nationwide increase in consumption intensity was observed Fig 4 raising concerns about microbial resistance [55]. In Brazil, the proportion of penicillin consumption during the study period was 20.1% in 2020, compared to 33.6% in 2018. Conversely, the highest relative consumption of macrolides was observed in 2020 (34.0%), and the lowest in 2018 (24.1%). It’s worth noting that international antibiotic consumption standards generally recommend higher usage of penicillins (J01C) than macrolides or any other class due to their lower potential to propagate or induce resistance [55,56].

An evaluative analysis among the ESAC-Net and WHO Regional Office for Europe [29] during 2014–2018 revealed that in 97% of ESAC-Net countries, penicillin consumption ranged from 24.3% to 65.9%, while in 67% of the 15 WHO AMC Network countries, penicillin consumption ranged from 14.2% to 40.2%. China also reported higher penicillin consumption (40.5%) than macrolides (10.2%) [28].

Despite their precarious position concerning macrolides, the decrease in penicillin consumption observed during the study years may be partly linked to the phenomenon of global shortages of active pharmaceutical ingredients, especially benzylpenicillin (J01CE), which began in 2014 [57]. Due to residual stock and free provision in public sector pharmacies, the observed increase in consumption in retail dispensing only had an impact from 2016 onwards, when 60% of Brazilian states faced extreme shortages in public dispensaries, which aggravated an increase in congenital syphilis cases in the country [58]. As an alternative treatment for syphilis, the Ministry of Health proposed the use of doxycycline (J01AA02 – a tetracycline), leading to an increase in the consumption from 2015 to 2016 (Fig 3). Ceftriaxone (J01DD04), a cephalosporine that peaked in 2017, was also recommended [59].

The atypical consumption of Other antibacterials (J01X), specifically nitrofurantoin (J01XE01) — a synthetic antibiotic commonly used to treat urinary tract infections — is noteworthy, concentrated in Santa Catarina in 2020. Although an increase in the incidence of these types of infections is expected during summer [60], studies have indicated that there was an increase in urological conditions due to restricted access to health services during the COVID-19 pandemic and the effects of long COVID [61,62]. However, the change in health determinants during COVID-19 is insufficient to justify this result. The SNGPC lacks a mechanism to flag outliers or prevent dispensing quantities well above treatment standards and guidelines for individual patients. According to Ferreira et al., the quantities of vials and boxes ranged from 1 to 536 units during the studied period, suggesting a significant error in the records of quantities dispensed [17].

SNGPC does have problems. However, information is fed into the system by a pharmacist. A more effective contribution would be expected from the pharmacist in ensuring the integrity of the data recorded in the system. According to Mubarak et al. [63], significant gaps in the training of these professionals may hinder the rational use of antibiotics. These gaps, however, should not only address clinical aspects, but also adequate data management, which can contribute to more accurate situational diagnoses of consumption, an essential tool in implementing new and timely practices in the safe use of antibiotics.

Other studies have used systemic antibiotic dispensing data from SNGPC, but each employed a different approach. Caetano et al. [64] employed a sample strategy, whereas our study utilized all processed dispensing records for individuals aged 15 years and above. Lopes et al. [16] focused on a shorter time span with a regional approach. Furthermore, neither added details on data processing, which, given the nature of the SNGPC records discussed in our previous work [17], is deemed critical for adequate data interpretation.

### Study limitations

The data used in this study comes from systemic antibiotic dispensing records for population >= 15 years obtained from the SNGPC. Unfortunately, dispensing information in the public sector is not centralized or publicly available. Ideally, the private and public sector results should be consolidated and analyzed alongside epidemiological information and antimicrobial resistance indicators.

Additionally, it’s important to understand that the SNGPC allows for retroactive updating. This means that a pharmacy may have performed a dispensing operation in a given month/year in the past, so the results found here may differ slightly in subsequent analyses. In order to ensure data integrity, the dates of file downloads were systematically recorded. Nonetheless, data stability tends to increase with time, and our data was processed almost two years after the last year of the extraction period.

The dataset used in this study underwent meticulous pre-processing. Approximately 28.5% of the total data extracted from the System was excluded during this process, due to the established inclusion criteria. Two researchers independently verified data against data dictionaries, selecting systemic antibiotic records with complete information on strength, route of administration, and dosage form. However, our analysis was centered exclusively on community dispensing and did not include dispensing performed in health facilities (hospitals and clinics), because those are absent from SNGPC. The lack of ICD-10 information made it difficult to link variation in consumption to disease profiles, save for COVID-19, for which the literature substantiated some consumption trends.

Despite these limitations, it’s important to emphasize that this study serves as an important standpoint for research into factors that influence the consumption of J01 in Brazil.

### Recommendations

The study shows that the SNGPC serves as a valuable source of publicly accessible and unrestricted data to support researchers and health managers in studies on antibiotic consumption. However, as our previous study demonstrated, inconsistencies in the data may obscure the true consumption patterns. In this sense, careful study design and support from epidemiological and sound administrative data can complement information to clarify consumption trends.

We recommend that ANVISA closely monitor data quality and provide protection mechanisms to minimize possible information errors, especially in the overall quantity, spatial, and temporal distributions of dispensed medicines. Additionally, we suggest that Brazilian states prepare annual consumption reports as a useful surveillance tool. This would support prompt interventions in high-risk cases of possible microbial resistance at the community level.

## Conclusions

The results provide valuable insights into national trends in antibiotic consumption during the study period, allowing for comparisons of dispensing practices across different states. Each state’s profile can serve as a basis for local health managers to analyze and interpret the data, thereby fostering the development of new strategies to monitor the use of J01 antibiotics.

While socioeconomic factors, population size, and pharmacy availability can influence antibiotic consumption, they do not guarantee its rational use. We can infer that the high doses consumed (DID) in certain states, which are less populous or have lower incomes, are likely more related to prescribing practices or the quality of recorded data than to the factors mentioned above.

This analysis did not include the dispensing services from SUS pharmacies. However, consumption, supply, and shortages in SUS have the power to determine trends in private services. Notably, this was the case with penicillin.

Over the study period, macrolide consumption consistently increased across all states. This finding raises concerns about conditions that may promote antibiotic resistance and highlights a critical area for intervention in clinical practice and pharmacotherapeutic monitoring.

We hope this research serves as a foundation for discussions on new surveillance and monitoring methods regarding the rational use of J01 antibiotics at the community level.
